# A study of mental health of EMS personnel during COVID-19 (A qualitative study)

**DOI:** 10.3389/fpubh.2025.1497197

**Published:** 2025-07-03

**Authors:** Alireza Sanatkhah, Ali Rahmani Ravari, Reza Ghanizade Shoabjarei

**Affiliations:** Department of Sociology, Kerman Branch, Islamic Azad University, Kerman, Iran

**Keywords:** corona virus, mental health, EMS, grounded theory, COVID-19

## Abstract

**Background:**

Medical staff that was under a lot of pressure and had insufficient resources and facilities during the outbreak of COVID-19. Accordingly, the present study attempts to gain a wider understanding of the reasons and factors affecting the mental health of Birjand EMS personnel during COVID-19.

**Methodology:**

The Grounded Theory method was used in the present study. Using in-depth and free interview technique according to this theory and also, by using targeted sampling, based on the inclusion criteria, the required data was collected and adjusted from 25 personnel of this organization. The samples were selected according to theoretical saturation and using the purposive sampling method. A semi-structured interview was used as a data collection tool. Additionally, three methods of control or validation by members, analytical comparisons and the use of audit techniques were used so as to achieve the criterion of validity.

**Results:**

Based on the results of the present study, stressful work and social environments, the risk of mistakes and occupational accidents, as well as physical disorders are known as the most important causal conditions. Undesirable salary and benefits, perceived social dignity and trust, and perceived social hope have been proposed as intervening factors. Distrust in management and cultural-educational infrastructural defects are mentioned as background conditions and reformatory measures of the government, increase in the level of public awareness, increase in the motivation of personnel, improvement of work and professional skills as action-interactions, behavioral-functional reaction and physical-psychological symptoms have been identified as outcomes.

**Conclusion:**

The mental health of personnel is formed under the influence of internal and external organizational (social) factors. The most important factor that affect it is management performance in society.

## 1 Background

In December 2019, the spread of a viral disease was reported in Wuhan, China. This disease was caused by a new type of genetically modified virus from Coronaviridae, called SARS CoV-2, named COVID-19 (1), p. 367. Unfortunately, due to its very high transmission power, this virus quickly spread throughout the world and infected all the countries in a short time (2), p. 194.

**Figure 1:**
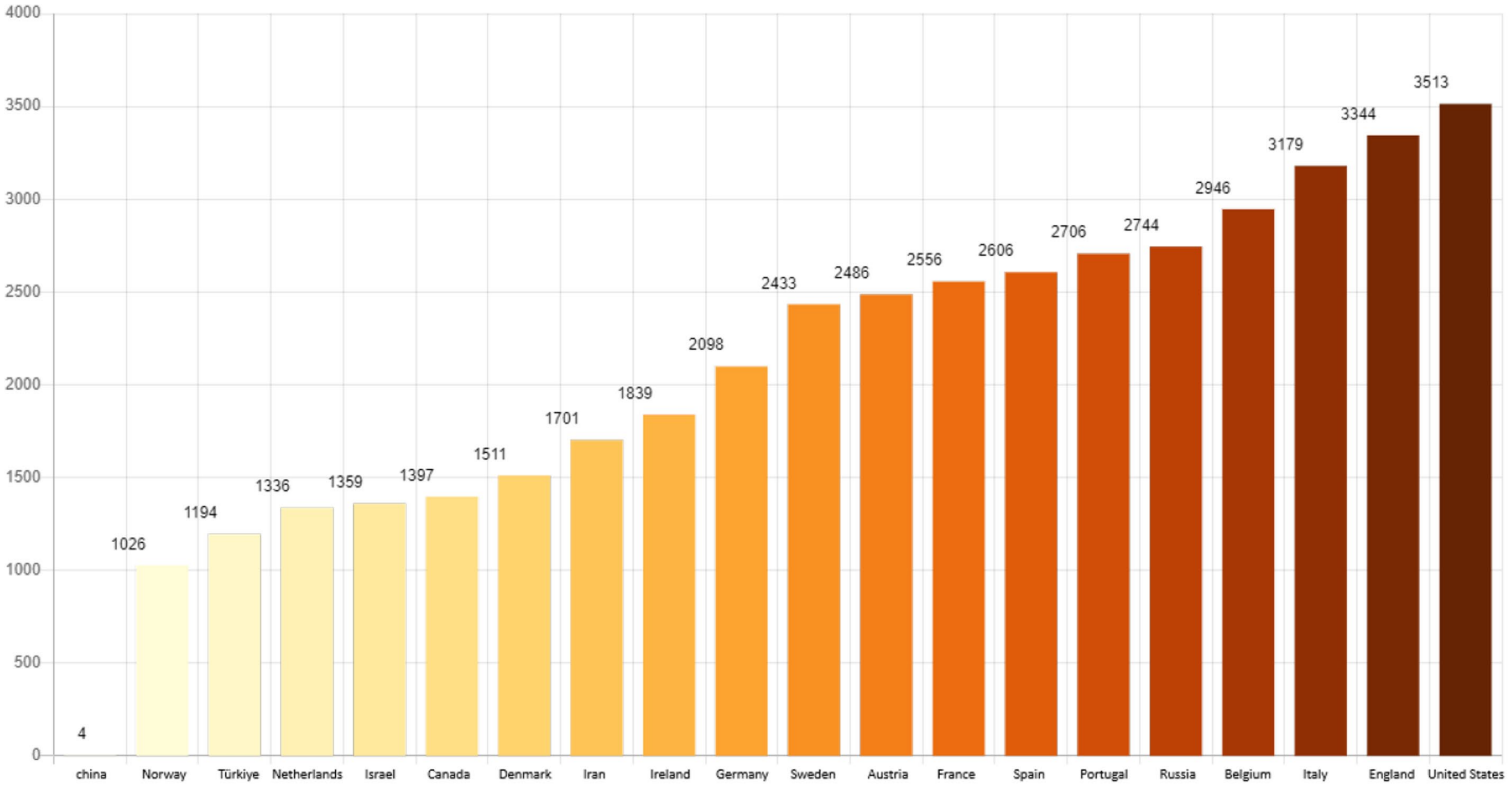
Number of deaths per million people.

According to the World Health Organization, so far, the total number of cases diagnosed with COVID-19 is 695,781,740, from among which 627,110,498 people have recovered and unfortunately 6,919,573 people have died. The number of deaths per 1 million people shows that the United States, England, Italy, Belgium and Russia have the highest number of deaths and Iran ranks 13th in the world (Figure 1).^1^

In Iran, this virus has had a lot of psychological effects on the citizens, especially the medical staff who have been in direct contact with COVID-19 patients. According to global health experts, health is complete physical, mental, and social wellbeing and, not just the absence of disease and the personal and social aspects of health and mental health.

According to the data collected from reliable sources, mental health plays an important role in people’s social, occupational and academic performance. For instance, World Health Organization states that appropriate work can help maintain mental health, and healthy and safe work environments are not only a fundamental right, but also reduce the possibility of tension and conflict in the workplace and help maintain the workforce, work performance and productivity (3). Moreover, the Centers for Disease Control and Prevention (CDC) emphasizes that mental health problems can negatively affect job performance, work interactions, relationships with coworkers, physical abilities and daily functioning (4). These data demonstrate that mental health is directly related to quality of life and people’s ability to participate effectively in society.

Studies show that major depressive disorder is common in Europe and the United States. For example, a study in Europe showed that the prevalence of depressive disorders was 10.05% (95% Cl 7.80–12.85) in women and 6.61% (95% Cl 4.92–8.83) in men. The study also found significant differences in the prevalence of depressive disorders across study sites, and it grouped centers into three categories: high prevalence (urban Ireland and urban UK), low prevalence (urban Spain) and moderate prevalence (other sites) (5). These data verify that depressive disorder is a very common disease in Europe and there are significant differences in its prevalence across different places.

Several studies have been conducted on the psychological effects of COVID-19 pandemic on medical staff. For instance, an international study demonstrated that 67% of health workers suffered from moderate to very high psychological stress (6). In addition, a quick review of studies showed that nurses may be more at risk of negative outcomes of mental health compared to other health workers (7). These studies highlight that supporting the mental health of medical staff should be prioritized and also, planning to improve the quality of their care can help improve job performance and the quality of medical and nursing care.

In scrutinizing the long-term effects of the COVID-19 pandemic on five mental and psychological disorders, Zhu et al. (8) addressed the long-term and negative impact of the epidemic environment on people’s mental and psychological conditions. In the study of Maliwichi et al. (9), which scrutinized the effect of COVID-19 pandemic on the mental health of health workers in Malawi, after regression analysis, it has been demonstrated that rates of depression, anxiety and PTSD among health workers in urban referral hospitals are significantly higher than those in regional hospitals. Qualitative findings indicated emotional stresses, impact on work and personal life, and experiences of stigma and discrimination having been faced by health workers. In their study entitled the impact of the primary outbreak of COVID-19 on the mental health of young people, Wiedemann et al. (10) demonstrated that outbreak of COVID-19 led to significant deviations from existing pathways related to mental health compared to the levels expected in the last 7 years. About a third of young people reported experiencing clinically significant depression (28.8%) or anxiety (27.6%) according to the current guidelines of NHS.

Rodolfo Rossi et al. (11), in a study of 1,379 frontline and second-line healthcare workers in Italy, demonstrated that 49.38% of participants experienced symptoms of post-traumatic stress disorder (PTSS), 24.73% suffered from depression, 19.80% experienced anxiety, and 8.27% reported insomnia. Additionally, 21.90% of these workers experienced high levels of stress. This study highlights that working on the frontline and directly encountering COVID-19 patients is associated with an increased risk of psychological problems, including PTSS and depression. Other factors, such as being younger and female, also increased the likelihood of mental health issues. Accordingly, the researchers emphasized the necessity of psychological interventions to prevent long-term effects on the mental health of healthcare workers.

Lai et al. (12), in a cross-sectional study involving 1,257 healthcare workers in China, reported that 50.4% of participants experienced symptoms of depression, 44.6% reported anxiety, 34.0% suffered from insomnia, and 71.5% experienced symptoms of stress. This study indicated that women, nurses, frontline workers, and those working in Wuhan were more likely to face mental health issues. Multivariable regression analysis showed that working on the frontline was associated with increased risks of depression (OR = 1.52), anxiety (OR = 1.57), insomnia (OR = 2.97), and stress (OR = 1.60). The researchers highlighted the importance of implementing psychological interventions to support the mental health of healthcare workers.

Sialakis et al. (13), in a systematic review and meta-analysis of 14 cross-sectional studies involving 7,780 healthcare workers, reported a prevalence of depression of 33.8% and anxiety of 41.3% among healthcare workers. This study demonstrated that the psychological pressure caused by the COVID-19 pandemic had a significant impact on the mental health of healthcare workers. The authors emphasized the necessity of continuous mental health monitoring and psychological interventions to reduce stress and improve the quality of life for healthcare workers.

Ghahramani et al. (14), in a systematic review and meta-analysis of 29 studies, reported that the prevalence of depression among healthcare workers was 36%, while the prevalence of anxiety was 47%. The study showed that insomnia, anxiety, and stress caused by the COVID-19 pandemic significantly affected the mental health of healthcare workers. The researchers highlighted the need for effective interventions to improve the mental health of this group and recommended special attention be given to physicians, nurses, and older healthcare workers.

In their study entitled the effect of COVID-19 on psychological adjustment problems, Şanlı et al. (15) showed that the effect of COVID-19 significantly predicts levels of meaning in life, resilience and psychological adjustment problems. Also, meaning in life and resilience emerged as significant predictors of psychological adjustment problems.

On the other hand, owing to the fact that most of the studies about mental health have been conducted using quantitative and survey methods, it can be said that one of the necessities related to the phenomenon of mental health in Iran, which requires study and data analysis and has not been addressed in previous researches, is discovering the semantic understanding and interpretation of people (EMS personnel) of the studied phenomenon and the way they give meaning to the phenomenon of mental health, so as to provide a detailed and deep description of this understanding and interpretation. Thus, the present study, by considering the EMS personnel as the main actors in the field of this phenomenon and focusing on internal and external issues, as well as the socio-cultural structure of their work environment, as a reality that is understood and interpreted by them, has attempted to discover the mental health phenomenon of personnel and its consequences during the COVID-19 pandemic, and also, provide a comprehensive analysis and interpretation of it in the context of the study, by using the interpretative approach and analyzing the semantic system of these people through delving into their inner experience.

Therefore, the main question of the research is “what is the understanding and interpretation of EMS personnel of Birjan about internal and external factors affecting the mental health of personnel during the Covid-19 pandemic?” The present study also addresses the question of “how the consequences of this virus on the mental health of the personnel is understood and interpreted by them?”

According to the latest census, Birjand has a population of more than 230,000 people and an area of 3,949 Sqm. It is located in the south of Khorasan province with a common border with Afghanistan for about 140 km, which has led to the entrance of several immigrants to Iran. Thus, health and health problems are affected by their arrival, which has increased the activity of the medical staff of this city and a scientific study must be conducted on it.

The innovation brought by the present study, compared to the previous researches, is that by considering the internal and external environment in the hospital and EMS department, it scrutinizes the occupational and social stressors, the risk of mistakes and the occurrence of occupational accidents as the most important causal factors, as well as distrust in management and cultural-educational infrastructural defects as background factors, and discovers intervening factors, consequences and interactive strategies of EMS personnel in dealing with mental illnesses and finally, opens a new horizon in scientific research during COVID-19.

The most important questions of the study are:

- What are the background factors that have affected the mental health of EMS personnel of Birjand during the outbreak of COVID-19?
- What are the causal factors that have affected the mental health of EMS personnel of Birjand during the outbreak of COVID-19?
- What are the factors that have interfered with the causal and background factors in the mental health process of EMS personnel of Birjand during the outbreak of COVID-19?
- What are the strategies (action-interactions) that have been used to improve the mental health of EMS personnel of Birjand during the outbreak of COVID-19?
- What are the consequences of COVID-19 on the EMS personnel of Birjand during the outbreak of COVID-19?

## 2 Methods

The preset study uses a qualitative and Grounded Theory method. The researcher systematically seeks to develop a theory to explain the process, action and interaction regarding the mental health of EMS personnel during the outbreak of COVID-19 in Birjand, discovers the meanings in the data through this systematic approach and also, during the coding process, puts the discovered meanings in the categorical containers. Finally, he presents a paradigmatic model and a theory limited to a specific reality at higher levels (16), p. 152.

When it comes to the qualitative sampling method, initially, given the sensitivity of the research topic and the difficulty in accessing participants, purposive sampling based on inclusion criteria was employed. Subsequently, theoretical sampling guided the continuation of the research process to support the development of the grounded theory. The research progressed such that theoretical saturation was achieved after interviewing the twentieth participant. However, to ensure that no new categories emerged, interviews were continued up to the twenty-fifth participant.

The criterion for theoretical saturation was based on the observation that after the twentieth interview, participants began to provide similar and repetitive responses, with no significant new findings. This criterion is widely recognized in qualitative research literature as a standard indicator for determining data adequacy. In assessing data repetition, whenever a code emerged from the data categorization, we compared it with previous codes to ensure that this concept, and the code assigned to it, had not been repeated before. For this reason, the non-emergence of new and meaningful information was the criterion for theoretical saturation. For example, in interviews with the twentieth to the twenty-third participants regarding intervening conditions, repetitive responses similar to other provided answers and codes were obtained, and these repetitive responses continued through the twenty-fifth interview (Table 1).

**Table 1:** Sample repetitive codes indicating theoretical saturation of the data.

Concepts	Initial code
Interview question: Describe the conditions at the workplace environment of the EMS (115) base and the emergency patient setting that intervene in the mental health of nurses (either disrupting or strengthening this relationship).	
“In the current unstable situation, it is difficult to balance income and expenses, and we cannot plan based on our salaries.” (P20)	Issues related to salary and benefits
“Delayed payments and wage arrears are among the most significant economic challenges we have faced since the COVID-19 pandemic.” (P21)	Issues related to salary and benefits
“Any person performs better when they have proper welfare conditions. EMS personnel, like many other employees, have zero benefits. We lack welfare facilities and accommodations. In the EMS system, there are no travel hotels for EMS staff, while employees in other executive organizations enjoy many more facilities and benefits. When our personnel compare their conditions with those of other organizations, it affects their mental well-being.” (P23)	Welfare and livelihood challenges
“Low payments and delays in salary payments are constant issues.” (P24)	Issues related to salary and benefits
“Despite the fact that performance-based bonuses are meant for operational staff, I see that they are often more substantial for administrative personnel.” (P25)	Unfavorable financial incentives
“When I first started working at this station, I thought I was strong and that these patients would not affect me. But now, my tolerance has decreased. I’ve had sleep problems for years due to heavy shifts. If we have a dangerous patient during dispatch, I constantly worry about it at home and sometimes call the center several times. I’ve developed back pain—my doctor says it’s a herniated disc—because we have to carry patients from upper floors and difficult-to-access areas using stretchers. I’ve also developed severe headaches and always feel exhausted.” (P25)	Physical health impairments Heavy work shifts

### 2.1 Participants

The participants in this study included 7 women and 18 men from the pre-hospital emergency medical services (EMS) personnel in Birjand during 2020. The total number of EMS personnel in Birjand was 31, comprising 7 women and 24 men. During the sampling process, the gender composition was determined based on active roles in the emergency department (excluding administrative staff such as record-keeping and office management) to ensure that the final sample accurately represented those directly engaged in the emergency setting.

Despite the unequal gender distribution (7 women and 18 men), this composition reflects the actual working conditions of the EMS environment, and the perspectives of both genders were incorporated into the analysis of the study’s main themes. This limitation is discussed as a point for future research in the concluding section of the study.

### 2.2 Data collection

The required data were collected using free and in-depth interview technique, which were finally analyzed using constant comparisons and theoretical coding (open, axial, selective coding).

Coding refers to assigning the closest concept to the smallest meaningful unit of each part of the collected data. In general, each concept has dimensions, and each dimension contains a range. In *open coding*, the researcher reads each interview transcript several times and records the main sentences as codes. Then, conceptually similar codes are grouped into categories. In *axial coding*, the initial codes and categories generated during open coding are compared, and similar items are merged. Categories that are related to each other are aligned along a common axis. The focus of this stage is on the conditions that lead to the phenomenon under study. During axial coding, the researcher deals with concepts that are also included in this study. At this stage, the paradigm model, including the phenomenon, causal conditions, core category, intervening conditions, strategies, consequences, and context, is identified.

**Figure 2:**
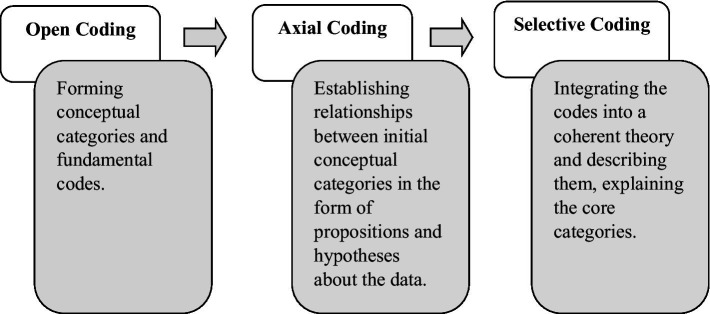
Stages of coding.

*Selective coding* is performed by choosing and identifying the core variable. In this stage, the researcher focuses on the process underlying the data and attempts to determine which category or variable is most frequently repeated and has the ability to relate other variables together. After axial coding, the final stage, selective coding, is conducted. At this point, the theory has become relatively robust, and the researcher deals with a limited number of categories, possibly making some theoretical refinements (Figure 2).

Memo writing in this study was carried out in such a way that the researcher continuously documented their thoughts and interpretations during the process of data collection. This is because the continuous and accurate documentation of data plays a significant role in the success of the research.

Theory writing and development: At this stage, the major categories were linked together within the framework of a paradigmatic model (also referred to as the contextual model), centered around the core category. The integration of categories involved the process of relating and connecting the major categories around a central concept, leading to the refinement and articulation of the theoretical constructs.

It is worth noting that the final contextual model must be able to explain under what conditions the studied phenomenon emerged, what mechanisms and interactions were involved, and what consequences or outcomes resulted from it. At this point, the researcher’s understanding of the study becomes clearer than ever, and their final task is to convey this understanding to others.

With the help of the constructed theory, it becomes possible to formulate hypotheses that can be tested in future studies, since the ultimate goal of this method is theory generation, not hypothesis testing.

### 2.3 Quality evaluation criteria (validity and trustworthiness)

Credibility: Credibility refers to the extent to which the findings of a grounded theory study are acceptable and trustworthy. This depends largely on the collection of authentic and accurate data. One of the key strategies for ensuring credibility in this study was long-term engagement during data collection. The researcher made a significant time investment to fulfill this criterion. In addition, the data analysis process was reviewed with the assistance of three university faculty members (two with PhDs in sociology and one with a PhD in crisis management), all of whom were experienced in qualitative research and had conducted reputable studies in this field. These experts were given access to the research process to review and provide feedback on various stages.

Dependability: Dependability in qualitative research is achieved when other researchers can clearly follow the research path and the steps taken by the investigator. Providing a precise record and report of the research process and decision-making enhances dependability. For this reason, the researcher carefully observed and documented the interview procedures and the coding and categorization process in detail.

Additionally, to increase dependability, several measures were taken: the use of semi-structured interviews, self-awareness regarding the researcher’s value system and beliefs to minimize bias toward participants and data collection, and post-interview reflection to document personal impressions and reactions. The researcher also strived to conduct interviews professionally, avoiding unnecessary probing into participants’ personal experiences.

An important effort was also made to avoid falling into the trap of premature theoretical saturation. Based on the researcher’s experience, some qualitative researchers, due to a lack of skill in conducting deep interviews, make early judgments about data repetition and similarity, which leads to premature claims of saturation.

Finally, the research findings were submitted for review by subject matter experts (two PhDs in sociology and one PhD in management) who were knowledgeable in the topic area, and necessary revisions were made based on their feedback.

Confirmability: Confirmability refers to the stability and consistency of the data over time and under appropriate conditions. Participant review, external audits, prolonged engagement, persistent observation, and data triangulation are among the strategies used to ensure credibility and confirmability. To achieve this, the research findings were shared with three participants who had greater knowledge and familiarity with the research topic so they could review the results. Additionally, the technique of “blind coding” was employed during the data coding process. This involved assigning parts of the dataset to independent researchers who were not members of the research team, to ensure objectivity in coding and interpretation.

Transferability: Transferability refers to the extent to which the findings can be generalized or applied to other groups or similar settings. It indicates that if other individuals from the same or similar groups—who were not part of the original study—read the findings, they would see a reasonable degree of similarity between the research results and their own experiences. At this stage, the findings were shared with two individuals who had experiences similar to those of the participants but had not taken part in the study. Their feedback was used to compare the results with real-world experiences, helping to demonstrate a reflection of reality within the research.

The details of the research participants are mentioned in Table 2.

**Table 2:** Details of participants in the research.

Row	Name	Gender	Age	Marital status	Employment history	Level of education
1	Mohsen	Male	28	Single	5	Bachelor of Emergency Medical Care
2	Mohammadreza	Male	33	Married	10	Bachelor of Emergency Medical Care
3	Zahra	Female	30	Single	7	Bachelor of Nursing
4	Musa	Male	37	Married	12	Bachelor of Emergency Medical Care
5	Mahdi	Male	37	Married	14	Bachelor of Emergency Medical Care
6	Farnaz	Female	39	Married	15	Associate of Emergency
7	Fatemeh	Female	35	Married	13	Bachelor of Emergency Nursing
8	Elham	Female	37	Married	13	Medical Emergency
9	Maliheh	Female	38	Married	15	Bachelor of Nursing
10	Mostafa	Male	32	Single	10	Bachelor of Emergency Medical Care
11	Mohammad Ali	Male	34	Married	13	Bachelor of Emergency Medical Care
12	Mohammdreza	Male	25	Single	3	Associate of Emergency
13	Ali	Male	41	Married	17	Master of Management
14	Ismaeil	Male	35	Married	12	Associate of Emergency
15	Mojtaba	Male	37	Married	14	Master of Management
16	Mojtaba	Male	45	Married	21	Bachelor of Emergency Medical Care
17	Fatemeh	Female	40	Married	16	Bachelor of Nursing
18	Ruhollah	Male	30	Married	8	Bachelor of Emergency Medical Care
19	Toktam	Female	27	Married	5	Bachelor of Nursing
20	Omid	Male	35	Married	11	Physician
21	Mohammad	Male	28	Married	7	Associate of Emergency
22	Mahdi	Male	37	Married	14	Bachelor of Emergency Medical Care
23	Akbar	Male	26	Married	4	Associate of Emergency
24	Alireza	Male	45	Married	25	Master of Psychology
25	Qasem	Male	32	Married	7	Master of Nursing

### 2.4 Data analysis

The present study uses the conventional analysis method proposed by Graneheim and Lundman (17). Qualitative analysis of content is a research method for conceptual interpretation of textual data through systematic classification, coding and development of themes or design of known models in order to obtain rich and deep information about the studied phenomenon (18).

Accordingly, immediately after each interview, the first author (FM) implemented the recorded content. In the reading phase, the implemented versions were carefully read line by line and important paragraphs were marked. Words, sentences, or paragraphs that were relevant to psychological challenges in EMS were also selected as semantic units. Each semantic unit was assigned a code. In the next step, the second author (BT) reviewed the implemented versions and confirmed the meanings and codes of units. Codes that were similar and homogeneous were combined and formed categories. To verify the reliability of the codes, the researchers reviewed the categories and compared them with the original data. Finally, after reflecting and comparing the categories in several joint meetings, the research team extracted the obtained themes (19), p. 3.

In some categories, quantitative data have been presented. Although this is a qualitative study and its primary aim is not statistical analysis but rather an in-depth understanding of experiences, frequency tables have been included solely to indicate the relative prevalence of themes, not for statistical inference. Nevertheless, care has been taken to ensure the accurate presentation of descriptive data (frequencies).

## 3 Results

The aim of the present study is scrutinizing the semantic understanding and interpretation of the EMS personnel of Birjand about the consequences of corona disease on the mental health of personnel based on the Grounded Theory. The coding process has been conducted in three stages (open, axial, selective coding) and sentence by sentence. With the emergence of the concepts resulting from line-by-line coding, the study entered the axial coding stage and the concepts were linked to the subcategories related to the data, then, the subcategories were linked to the main categories. In the following of the axial coding process, the general categories obtained during the open coding process were adjusted to the framework of the paradigm model. This model addresses the conditions, backgrounds, strategies and consequences of the COVID-19 on the mental health of EMS personnel. Finally, in the selective coding stage, the core category was selected and linked regularly with other categories using the storytelling technique. The main categories and subcategories obtained from the research flow are as follows.

### 3.1 Causal conditions

Causal conditions are those that affect the phenomena. In this research, causal conditions are the factors that cause disturbances in the mental health of EMS personnel (20), p. 117 (Table 3).

**Table 3:** Causal conditions, concepts, main categories and subcategories.

Concepts	Subcategory	Frequency	Relative frequency percentage	Main category
Work pressure/Tense work environment/Heavy shifts (special shifts)/Unstable work environment (unstable conditions of contract staff)/Poor nutrition	Work Environment	10	40%	Stressful Work and Social Environments
Colleague understanding/Lack of interaction with colleagues/Personnel’s mental weakness/Colleagues’ withdrawal	Colleague Relations	9	36%	
Heavy missions/Delays in patient acceptance by doctors/Lack of time to reach the scene/Unnecessary calls (false missions)/Patient transport in difficult locations	Mission Issues	4	16%	
Family stress/Family and related incidents/Stress from family relationships/Tense family environment/Stressful economic problems/Lack of time for family/Stress from family and life problems	Life and Social Environment Stress	8	32%	
High crowding at the incident scene/Public interference in patient transfer/Crowding the scene/Taking photos of critically ill patients and deceased by public	Lack of Public Cooperation	10	40%	Risk of Mistakes and Occupational Accidents
Non-compliance with hygiene protocols/Lack of public awareness about COVID-19 hygiene/Public interference in missions	Non-compliance with Hygiene Protocols	4	16%	
Lack of awareness about EMS duties/Unreasonable expectations from the public/Poor interaction with patient companions	Lack of Awareness about EMS Duties	3	12%	
Gastrointestinal problems from job stress/Physical issues such as cardiovascular, nervous system, and skeletal problems (e.g., arthritis, disc issues, physical problems)	Personnel Physical Health	4	16%	Disruption in Physical Health
Sleep disorders, bad sleep, insomnia from shift work, loss of sleep balance, excessive fatigue	Personnel Sleep Patterns	6	24%	
Heavy shifts/Excessive fatigue from night shifts/Emergence of physical issues from overwork/Physical exhaustion	Nature of Shift Rotation	8	32%	
Encountering critically ill patients/Emergency surgeries/Dangerous accidents/Distressing scenes/Self-immolation and severe scenes/Horrific accidents	Psychological, Mental, and Emotional Triggers	6	24%	

This table illustrates the factors that directly affect the mental health of EMS personnel. The most significant impact is attributed to stressful occupational and social environments, including work pressure, heavy shifts, unfavorable workplace conditions, and interpersonal relationships among colleagues. For example, some participants reported experiencing psychological strain due to challenging missions and insufficient interaction with their coworkers.

Additionally, issues such as the lack of public cooperation and non-compliance with health protocols during the COVID-19 pandemic have exacerbated the risk of occupational mistakes and accidents, further intensifying the mental health challenges faced by EMS personnel.

In general, quantifying qualitative categories is not recommended in grounded theory research, as the goal of such studies is to gain a deep understanding of phenomena rather than to generalize findings to the entire population. However, to illustrate the relative importance and frequency of each extracted category, the data have been quantified to a limited extent. It should be noted that among the 25 participants, some respondents may have referred to multiple items in response to a single question; therefore, the total number of responses may exceed the number of participants.

One of the causative factors that affect the mental health of emergency personnel is “occupational and social stressful environments,” which are characterized by subcategories such as “work environment conditions,” “relationship with coworkers,” “mission problems” and “life and social environment stress.” Personnel No. 1 declares that “improving people’s well-being, people’s economic situation can make us feel good as well. High work pressure and long shifts due to the lack of personnel increase anxiety and stress (P1).” On the other hand, personnel No. 6 says “most of the time, owing to the discomfort, the patient treats the EMS personnel badly. Sometimes, these assaults have injured our coworkers, which has significantly affected the mental health of our coworkers (P6).” Personnel No. 3 complained about “unstable conditions and lack of job security for company personnel (P3).” From among other causative factors, we can refer to “the risk of mistakes and occupational accidents” which can be associated with sub-categories such as lack of public support, non-observance of health protocols by the people, and lack of awareness of the EMS duties among the people. Personnel No. 14 complained about the improper expectations of people from EMS personnel in the event of an accident and declared that “Sometimes we see that the patient’s companions behave aggressively and this affects our mental health a lot (P14).” Personnel No. 15 said: “People are not that much aware of the EMS and their improper expectations sometimes cause verbal and physical conflicts (P15).”

### 3.2 Underlying or background conditions

Background conditions are a specific set of conditions that gather at a specific time and place to create a set of conditions and issues that people respond to with their leaders. In the present study, after the interviews, it was found that it there are factors that affect the mental health of EMS personnel under the framework of background conditions (16), p. 152, quoted from (20), p. 117 (Table 4).

**Table 4:** Background conditions, concepts, main categories and subcategories.

Concepts	Subcategory	Main category	Frequency	Relative frequency percentage
Ineffective management system/Valuing patronage over merit in management/Political and party-based management instead of scientific management/Unnecessary rigidity	Ineptitude	Distrust in Management	13	53%
Lack of personnel recovery/Lack of managerial experience/Dishonesty of managers/Lack of financial support	Trial-and-Error Management Style			
Incorrect statistics/Slow vaccination process/Lack of transparency and honesty	Functional Weakness			
Lack of practical and hands-on training/Weak professional skills training/Insufficient knowledge about COVID-19/Lack of awareness among personnel regarding the virus	Educational Weakness	Cultural-Educational Infrastructure	8	33%
Lack of equipment/Shortage of ambulances and presence of old vehicles/Lack of medication/Absence of welfare facilities/Shortage of staff	Equipment Weakness			
Poor handling of patients and their companions/Unreasonable patient expectations/Patient aggression/Severe patient conditions/High mortality rates	Patient’s Physical and Mental State			
Lack of public responsibility/Failure to follow laws and restrictions/People’s lifestyle	Lack of Responsibility			

This table identifies the conditions that indirectly contribute to the mental health challenges faced by EMS personnel. One of the most significant categories discussed is “distrust in management,” which stems from factors such as incompetence, a trial-and-error approach to management, and functional weaknesses.

Additionally, weakness in cultural and educational infrastructure also encompasses a lack of practical and experiential training, leaving personnel unprepared to effectively handle real-world scenarios. This deficiency undermines their ability to confidently and competently respond to emergencies, exacerbating their psychological and professional challenges.

One of the areas affecting the mental health of emergency personnel can be called “distrust in management” which is characterized by subcategories such as “incompetence” and “trial and error management method” and “functional weakness.” According to the personnel No. 4 “In our society, the management system of healthcare is insufficient (P4)” and according to Personnel No. 17, “our work environment is not stable and we have various patients, patients who need oxygen and we are facing a shortage, it is a problem for us (P17).” Personnel No. 24 says “sometimes, the straw just breaks the camel’s back. I do not know what to do. Therefore, I may behave aggressively with my colleague, or my spouse or children. I cannot control myself, I really want to do this, but I do not know how (P24).”

### 3.3 Intervening conditions

Intervening conditions are those that facilitate or constrain the factors that influence the strategies. These conditions modify the causal factors (21), p. 35–1. In the present study, based on the interviews certain factors were identified that could intensify or weaken the mental health of EMS personnel. These factors, under the intervening conditions, are presented in Table 5.

**Table 5:** Intervening conditions: concepts, subcategories, and main categories.

Concepts	Subcategory	Main category	Frequency	Relative frequency percentage
Low salaries and benefits/Lack of incentives/No bonuses/Delayed payments and overtime/Discrepancy between salaries and inflation	Problems with Salaries and Benefits	Undesirable Salary and Benefit Conditions	22	88%
Severe economic problems for families/Inability to meet basic needs/Problems registering children in schools/High school fees/Severe economic pressures	Welfare and Living Problems			
Lack of benefits for nurses’ families/No welfare benefits compared to staff members/Material incentives/Inadequate incentive system for personnel	Poor Economic Incentives			
Social respect for personnel/Community respect for nurses/Valuing the nursing and emergency professions/Feeling valued in society	Social Status	Perceived Social Status and Trust	8	32%
Distrust of government institutions and organizations/Distrust of municipal authorities/Distrust of provincial authorities/Distrust in welfare services/Distrust in the passage of proper laws in parliament/Distrust of officials	Social Trust			
Participation in social activities/Participation in government rallies/Voting/Promoting candidates	Social Bond	Perceived Social Hope	6	24%
Family, friends, and community support against psychological pressures and incidents	Perceived Social Support			
Hope for future employment/Hope for improvements in social, political, health, and living conditions/Hope for better air quality and environmental conditions	Hope for Life			

This table addresses the factors that amplify or mitigate the impact of causal conditions. The most significant factor is the “unsatisfactory state of salary and benefits,” which includes financial and livelihood challenges as well as the absence of economic incentives. In addition, social status and hope for life serve as supportive variables that can alleviate the psychological pressures stemming from workplace conditions. For instance, participants highlighted the lack of financial support and the positive effects of social support in reducing stress.

One of the intervening factors that affect the mental health of EMS personnel is the “undesirable state of salary and benefits,” characterized by subcategories such as “salary and benefit issues,” “welfare and livelihood problems,” and “unsatisfactory economic incentives.” According to Personnel No. 9, “Colleagues wanted their salary to be paid on time, but unfortunately, I see delays in payments for clothing allowances, overtime, and very little economic support in the workplace. We do not receive any bonuses, New Year bonus, or travel benefits (P9).” Personnel No. 12 says, “Salary and benefits are low, and due to the COVID-19 situation, no bonuses or overtime allowances were paid (P12).”

Another intervening factor is “perceived social status and trust,” which is identified by subcategories such as “social status” and “social trust.” A key characteristic of this factor, according to Personnel Nos. 1, 3, 12, 11, 19, and 21, is the decline in trust within society, which is worsening by the day. Personnel No. 19 states, “Due to the COVID-19 pandemic, the involvement of personnel’s families, and the loss of many loved ones, trust declines, and mental health deteriorates (P19) (Table 6).”

**Table 6:** The importance of gender-based salaries and benefits.

Concepts	Women		Men	
	Frequency	Relative frequency percentage	Frequency	Relative frequency percentage
Improving public welfare and the economic conditions of society can positively impact our mental and emotional wellbeing. High workloads and long shifts, caused by staff shortages, lead to increased anxiety and stress (P1). Public welfare, better livelihoods, and improved economic conditions provide a foundation for maintaining mental health at the societal level. When the mental health of the community is at an optimal level, interactions with EMS personnel also improve (P10). Economic challenges, such as livelihood difficulties and delayed payment of salaries and benefits, are significant factors affecting the mental health of personnel. Delays in paying salaries, overtime, and mission-related compensation, which sometimes extend over several years, have been one of the most critical economic challenges following the COVID-19 era (P3 & P4). Low wages, the lack of bonuses, and unpaid overtime during the pandemic have had significant negative effects on the morale of staff (P9 & P10). The failure to pay arrears and provide incentive support, such as performance-based bonuses, certificates of appreciation, or other benefits, has reduced motivation among employees (P12, P13, P17). Additionally, the increased healthcare costs that became part of daily life after COVID-19 cannot be offset by low and fixed wages (P13). During the pandemic, EMS personnel did not receive the necessary support; even performance-based bonuses, which were intended for operational staff, were often allocated to administrative staff (P18 & P19). Low salaries and their impact on family livelihoods remain a serious concern for personnel, as family priorities are directly affected by unfavorable economic conditions (P21 & P22). Timely payment of salaries and benefits not only addresses livelihood needs but also enhances productivity and workplace morale. Conversely, payment delays significantly reduce the motivation and efficiency of staff (P25).	5	71.42%	17	94.4%

This table compares the significance of salary and benefits among EMS personnel based on gender. The results indicate that men (94.4%) are more likely than women (71.42%) to identify salary and benefits issues as a factor impacting their mental health.

Several factors may explain this difference:

1. Differences in livelihood responsibilities: Men, often considered the primary breadwinners of their families, experience greater financial pressure. Consequently, issues such as salary delays and unpaid benefits have a more pronounced impact on their mental health.
2. Livelihood and welfare challenges: Many men reported the financial strain caused by the high cost of living and delays in salary payments, leading to increased anxiety and diminished motivation.
3. Cultural and social expectations: In some societies, men are expected to play a greater supportive role in their families, which adds to their psychological burden.

On the other hand, while women also cited salary and benefits issues, their lower proportion may be attributed to factors such as reduced financial responsibilities within the family or a different perspective on job security.

This table underscores the critical role of improving salary and benefits, particularly for groups experiencing higher levels of stress, in alleviating anxiety and enhancing the mental wellbeing of personnel.

### 3.4 Strategies (actions-interactions)

Strategies or actions-interactions refer to specific actions or interactions that emerge from the main phenomenon. Strategies and actions are plans and actions that help design the model (21), p. 35. According to the participants, one of the strategies or actions-interactions is the government’s corrective measures, identified by the subcategories of “mass vaccination” and “improving economic conditions.” Participant No. 5 says, “Unfortunately, in the early stages of the COVID-19 pandemic, we did not perform well. There were the issues of vaccination, and sometimes inaccurate statistics that claimed the situation was normal while we were witnessing the high deaths due to COVID-19, but later on, the mass vaccination and government support measures for healthcare staff were evaluated as a desirable strategy.” Participant No. 8 also complains about “poor management at the macro level, especially during the early stages of the pandemic and the slow vaccination process.” Participant No. 13 says, “Once the importance of the issue and the spread of the disease became clear to the authorities, corrective measures were taken to provide necessary supplies such as masks and alcohol (P13) (Table 7).”

**Table 7:** Strategies (actions-interactions)—concepts, subcategories, and main categories.

Concepts	Sub-category	Main category
Providing facilities and medications—Vaccination during COVID-19 crisis—Controlling inflation	Public Vaccination—Economic Improvement	Government Corrective Actions
Public awareness of emergency duties/Promoting cooperation with emergency services on roads and at accident scenes/Promoting positive interactions with emergency personnel	Wide Introduction of Emergency Personnel	Increasing Public Awareness
Public awareness of COVID-19 crisis conditions/Awareness in patient transfer/First aid training for the public during crises	Extensive Information on COVID-19	
Incentive programs/Creating motivation/Personnel surveys/Thinking rooms/Presence of therapists at the station	More Attention to Personnel	Increasing Personnel Motivation
Maintaining calm—Understanding conditions/Coordination among personnel	Improving Personnel’s Personal Performance	Improving Personnel’s Professional Skills

### 3.5 Consequences

Whenever a specific strategy is implemented or not implemented in response to an issue or to manage or maintain a situation by an individual or group, the consequences of the phenomenon arise (21), pp. 1–35. The consequences of the COVID-19 pandemic on the mental health of EMS personnel are as follows: One of the consequences mentioned by the participants in the study is behavioral-performance reactions, characterized by subcategories such as “mental-psychological weakness of the personnel,” “conflicts and stress,” and “reduced performance.” When personnel experience psychological problems, they deliver the lowest quality of work, which may lead to negative interactions with patients’ companions outside the work environment (P2). When the mental health of the personnel is low, their decision-making ability in work situations decreases, and so does their job performance (P6) (Table 8).

**Table 8:** Consequences: concepts, subcategories, and main categories.

Concepts	Sub-category	Main category
Family conflicts/Reduced resilience/Verbal conflicts with others	Mental–Emotional Weakness of Personnel	Behavioral-Performance Reactions
Anger at work/Interactions with others/Job stress/Conflicts with others/Patients	Conflict and Stress	
Reduced quality of work and service delivery/Decreased concentration/Reduced decision-making ability/Poor service provision by personnel	Decreased Performance	
Physical functioning disorders/Depression/Severe stress/Severe anxiety/Restlessness and depression	Anxiety and Depression	Physical-Psychological Symptoms
Back pain from work pressure/Physical injuries of personnel/Physical problems/Exhaustion/Back and neck arthritis/Physical weakness	Physical Reaction	
Physical symptoms, anxiety, and insomnia/Poor sleep/Inadequate sleep/Stress during sleep/Disturbed dreams	Sleep Disorders	

### 3.6 Paradigmatic model of the study

**Figure 3:**
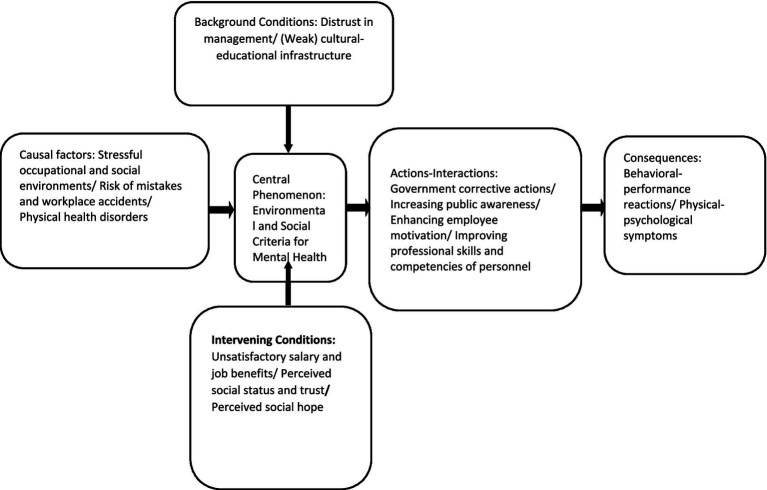
Paradigmatic model of grounded theory on the effects of COVID-19 on the mental health of EMS personnel.

The paradigmatic model of the research is indicative of the flow of processes and activities that occur within the context of the study. This model is one of the key components of the Grounded Theory method. The model consists of five parts: causal conditions, background conditions, intervening conditions, strategies, and consequences. The central phenomenon lies at the center of the model and all activities are organized around it. The paradigmatic model shows the flow of processes and activities shaped within the context of this phenomenon (Figure 3).

The paradigm model illustrates that causal conditions are those factors that influence the mental health of EMS personnel. Stressful occupational and social environments—which include workplace conditions (such as workload pressure, long shifts, and nutrition-related issues at the workplace), peer relationships (interpersonal dynamics among colleagues), mission-related problems (such as delays in reaching the scene, hospital admissions, etc.), and life and social stressors (family-related issues stemming from broader socio-economic conditions like financial hardship)—have a significant impact on the mental wellbeing of the personnel. In addition, lack of cooperation from the public (e.g., crowding at accident scenes) and non-compliance with health protocols increase the likelihood of errors and occupational incidents, further affecting their mental health. Physical health impairments are also considered one of the causal conditions influencing psychological wellbeing.

In this model, contextual conditions are identified as factors that provide the setting in which causal conditions exert their influence. Issues such as distrust toward management (including trial-and-error-based administration and poor managerial performance), as well as weaknesses in the cultural-educational infrastructure (including insufficient training in professional skills and lack of equipment and facilities), are recognized as key contextual factors affecting the mental health of EMS personnel.

Intervening conditions refer to factors that either facilitate or hinder the influence of causal conditions on strategies. These conditions may alleviate or modify the effects of causal factors. Such factors include: unfavorable employment benefits and salaries, perceived social status and trust (including public distrust in institutions and the perceived value and respect for healthcare staff in society), alongside perceived social hope (including social bonding or participation in community activities, hope for the future, and family and social support for EMS staff). These intervening conditions play an important role in shaping how causal and contextual conditions impact the mental health of personnel.

These factors guide the selection of strategies. The strategies include: government corrective actions, raising public awareness, enhancing staff motivation, and improving the professional and job-related skills of EMS personnel. If these strategies are effectively implemented, they may help reduce the consequences of the core phenomenon, such as behavioral-performance reactions and physical-psychological symptoms among personnel. However, if strategies are not properly implemented, the consequences may include psychological weakness, conflicts and stress, reduced performance, anxiety and depression, physical reactions, and sleep disorders.

## 4 Discussion and conclusion

During the outbreak of COVID-19, healthcare workers were more exposed to the virus than anyone else, and close contact with severely ill patients had a significant impact on their mental health. The aim of the present study is to understand and interpret the perspectives of EMS personnel of Birjand about the mental health consequences of COVID-19, based on Grounded Theory. In the present study, certain conditions have been identified as causal or contributing factors, one of the most important of which is “stressful work and social environments,” which is characterized by subcategories such as “work environment conditions,” “relationships with coworkers” “mission-related problems,” and “stress from individual and social life environments.” These results are in line with the findings of the study of Shahyad and Mohammadi (22) and the studies by Fathi et al. (23), Farahati (24), Rakhshan et al. (25). According to existing theories (conceptual framework), job stress can have negative effects on organizational variables such as individuals’ commitment and loyalty to the organization, as well as on individual organizational variables like job performance. AbuAlRub (26) identified the most significant workplace stressors as job demands (such as lack of control over tasks, task ambiguity), organizational factors (such as poor interpersonal communication, inappropriate managerial behaviors), economic and financial issues, conflicts between work roles and family responsibilities, aspects of career development and training (such as lack of opportunities for growth and promotion), and a poor organizational climate (such as a lack of managerial commitment to valuable personnel and the complexity of organizational communications). According to Hagen (27), stress is a key factor in organizational inefficiency, changing employees, absenteeism, reduced work quality and quantity, increased healthcare costs, and decreased job satisfaction. Based on the studies of Khaleghi and Latifi (28), according to the research participants, heavy workloads in the EMS department, along with challenging work shifts, poor nutrition conditions, and mission-related problems such as “refusal of hospitals to admit patients,” unnecessary calls, and limited time to reach the scene, were the major stressors which were associate with the mental health of EMS personnel. The close relationship between coworkers can reduce stress to an extent. However, the conditions in the emergency department during the COVID-19 pandemic have been in such a way that we have observed “poor interactions with colleagues” and weakened morale among the staff. These factors, combined with domestic and family issues such as stress within the family and a tense family atmosphere—largely rooted in economic difficulties—can intensify mental health disorders and negatively affect the mental wellbeing of the personnel. The risk of mistakes and occupational accidents is another factor related to the mental health of personnel. This issue is associated with subcategories such as the lack of public cooperation, the public’s failure to adhere to health protocols, and the lack of awareness about the duties of emergency services. The large crowds at accident scenes, such as traffic accidents—which were common both during and before the COVID-19 pandemic—the public’s interference in moving patients, and taking photos of critically ill patients by the people, along with the failure to observe health protocols during the pandemic, have increased the risk of occupational mistakes and accidents. On the other hand, factors such as “personnel’s sleep patterns,” the “nature of rotating work shifts,” and “mental, psychological, and emotional stressors” have caused disruptions in the physical health of EMS personnel during the COVID-19, which in turn has affected their mental health.

On the other hand, background conditions—which refer to a specific set of circumstances that come together in a particular time and place to create conditions and issues that people respond to with their strategies—include distrust in management. This is characterized by subcategories such as incompetence, trial-and-error management method, and functional weakness. According to the participants, management weakness is evident in issues such as the disregard for meritocracy, political and partisan management instead of scientific management, which has led to either wrong managerial decisions or delayed management actions. The trial-and-error management style is another issue within the management system, where management insists on experimenting rather than benefiting from scientific experiences, leading to functional weaknesses.

The cultural-educational infrastructure weakness, which is characterized by subcategories such as weak educational systems, lack of resources, the mental and physical condition of patients, and lack of responsibility, is another background condition that affects the mental health of EMS personnel.

On the other hand, the lack of practical and hands-on training, poor professional skills, and insufficient awareness among personnel in dealing with the virus create a suitable environment for reducing mental health. On the contrary, the lack of resources manifests in the shortage of adequate equipment, ambulances, medications, and similar necessities. In addition, the public’s lack of responsibility, lack of adhering to laws and restrictions, and their lifestyle choices have contributed to the rise in COVID-19 cases, which has placed a significant burden on emergency personnel.

Another factor affecting mental health is intervening conditions, which refer to circumstances that facilitate or limit the factors influencing strategies. These conditions change or modify causal factors. One of the most critical intervening factors is the undesirable state of salary and benefits, characterized by subcategories such as salary and benefit issues, welfare and livelihood problems, and inadequate economic incentives. These findings are in line with the results of the study of Alizadeh Fard and Saffarinia (29), Meyer et al. (2021), and Cai et al. (30).

Perceived Social Hope, which is characterized by subcategories such as social bonding, perceived social support, and life expectancy, is another intervening condition. According to Bandura’s theory (31), several factors affect the individuals’ mental health. Factors such as self-efficacy and social support help individuals cope with stressful situations, which will lead to less psychological harm. Self-efficacy beliefs significantly affect many aspects of personal functioning. Individuals with higher levels of self-efficacy are more likely to explore broader career opportunities, achieve greater professional success, set higher personal goals, and have better mental health. In this regard, the concept of resilience and effective coping strategies, as examined by Finstad et al. (32) as potential positive aspects of adapting to trauma in the workplace post-COVID-19, can serve as key factors in mitigating the negative effects of stress. Social hope and perceived social support, identified in their study, can be considered as the foundation for developing such resilience among emergency personnel. Furthermore, research such as the study by Wu et al. (33) indicates that resilience and the types of coping strategies individuals employ (especially when dealing with loss and grief caused by COVID-19) play a significant mediating role in their overall mental health outcomes. This highlights the importance of simultaneously addressing the reduction of stressors and strengthening individual and social resources for emergency personnel.

If we attribute strategies or actions-interactions to specific behaviors or interactions arising from the central phenomenon, then in the present study, the key strategies or actions-interactions can be categorized as governmental corrective actions, raising public awareness, increasing staff motivation, and improving personnel’s professional and work skills. Public vaccination and the improvement of economic conditions as part of the government’s corrective measures have successfully created favorable conditions for managing the COVID-19 virus. Measures like introducing emergency personnel to citizens, disseminating information about COVID-19, and paying more attention to the personnel can lead to improved personal performance, finally advancing better management of emergency personnel’s mental health.

The consequences of the COVID-19 pandemic on the mental health of EMS personnel are identified at two levels: behavioral-performance reactions and physical-psychological symptoms. Behavioral-performance consequences include mental-psychological weakening of the staff, conflict and stress, and reduced performance. Physical-psychological symptoms include anxiety, depression, physical reactions, and sleep disturbances during the COVID-19 pandemic. The prevalence of these negative mental health outcomes among frontline healthcare workers is a finding that has been widely acknowledged at an international level. As demonstrated in the comprehensive review by Butcher et al. (34), the pandemic has had profound and widespread impacts on the mental health of this occupational group worldwide. Finally, the concept of “cyclical functional erosion,” which arises from managerial performance and economic, social, and occupational challenges during the COVID-19 pandemic, is introduced as the main category.

### 4.1 Recommendations

The most important recommendations of the study are as follows:

1. Taking necessary measures for effective presence of EMS personnel in health-related crises through reducing occupational stress (by providing economic and job incentives to alleviate work and life pressures) and increasing the productivity and efficiency of human resources within the organization (by boosting morale with financial and motivational incentives, addressing housing and welfare issues, and aligning salaries with inflation rates).
2. Implementing preventive interventions for improved performance to ensure correct and better functioning of healthcare staff and EMS personnel by avoiding the endorsement of sycophantic behavior and relying on meritocracy for personnel promotions.
3. Providing adequate equipment to ensure the effective performance of emergency personnel in health-related crises by equipping them with suitable tools for their missions, including ambulances and necessary medical equipment.
4. Educating the public and providing essential training to citizens on how to interact with EMS personnel during incidents as a preventive measure in occupational social work.
5. Implementing health programs to improve personnel morale and address mental health issues by offering sports and wellness activities.
6. Ensuring the effective presence and performance of personnel in health-related crises by selecting managers based on meritocracy and their scientific and ethical qualifications.
7. Enhancing trust among the public and personnel by improving management practices and increasing welfare and health services as a preventive measure in occupational social work.

### 4.2 Ethical considerations

The present study was conducted according to the principles of the revised Helsinki Declaration, a statement of ethical principles that guide doctors and other participants in medical research involving human subjects. Prior to the interviews, all individuals were informed about the study’s objectives, the voluntary nature of participation, data collection methods, the reason for recording the interviews, the roles of the interviewer and participants, and the confidentiality and anonymity of the information. They were then asked to sign an informed consent form if they wanted to participate in the study. Participants were informed that they could withdraw from the study at any time.

### 4.3 Strengths of the study

This study is the first qualitative research in Iran which has been conducted with the aim of scrutinizing the mental health of EMS personnel during the COVID-19 pandemic. Gaining deeper insights into the challenges faced by EMS personnel can contribute to improving the quality of pre-hospital emergency care during epidemic crises.

### 4.4 Limitations

One limitation of this study is that the data were just gathered through individual interviews with EMS personnel of Birjand, Iran. Due to economic, cultural, and social differences between Iran and other countries, similar studies are recommended to be conducted in other countries. Moreover, future studies should be conducted with the aim of obtaining richer data by including physicians and other emergency care staff, patients, and their families in other locations, using a variety of data collection methods such as surveys.

The gender imbalance in the research sample can be considered one of the study’s limitations, as the experiences of women may not have been fully reflected. The small sample size of women does not allow for deeper analysis by the researcher, although efforts were made during sampling to ensure that the proportion of female to male staff in the emergency center matched the selected sample size.

Geographical limitation can also be identified as a significant constraint. For this reason, the study attempted to compare its findings with similar studies from other regions of Iran or around the world in the discussion and conclusion sections. However, despite the novelty of the research in Iran, there has been limited extensive investigation on this subject.

Another limitation of the study pertains to the generalizability of the findings. Since the research was conducted in the city of Birjand, Iran, and employed a qualitative methodology, while this approach provides in-depth insights, it lacks strong generalizability. To enhance the generalizability of the findings, quantitative and survey-based research is necessary.

## Data Availability

The datasets presented in this study can be found in online repositories. The names of the repository/repositories and accession number(s) can be found in the article/supplementary material.

## Footnotes

^1^ https://www.trt.net.tr/persian/covid19
